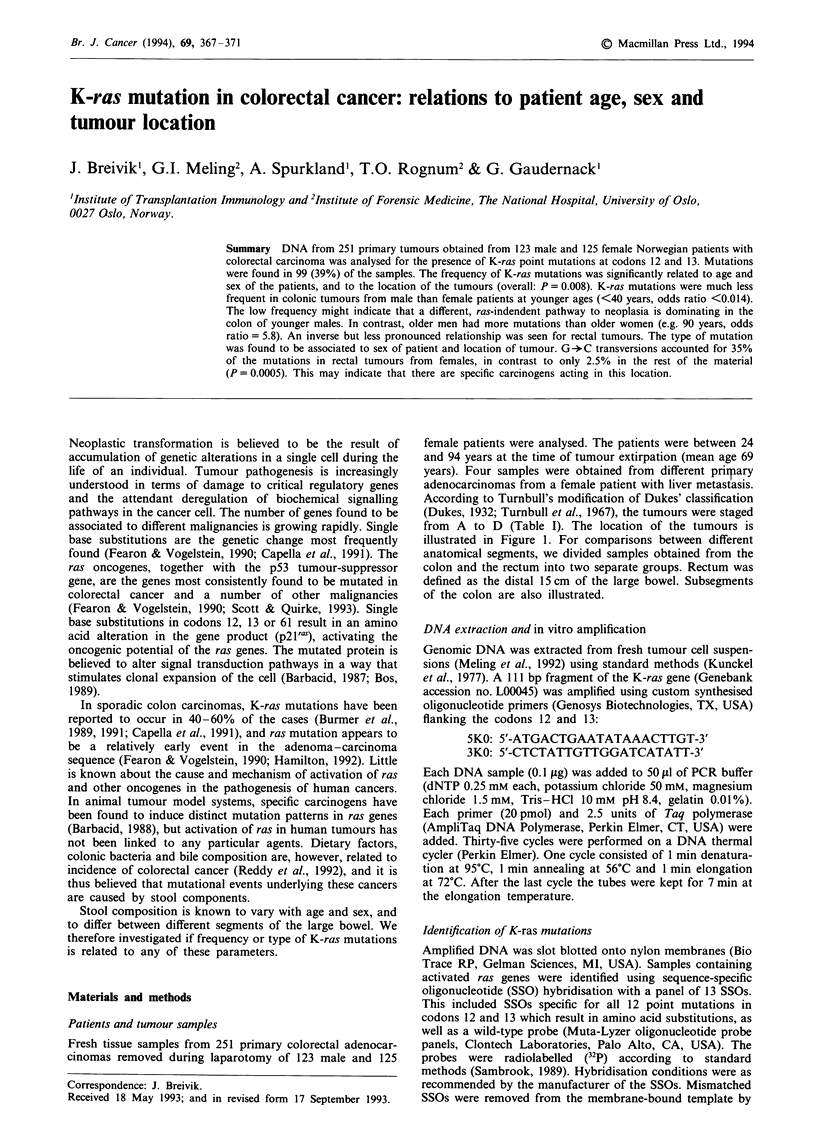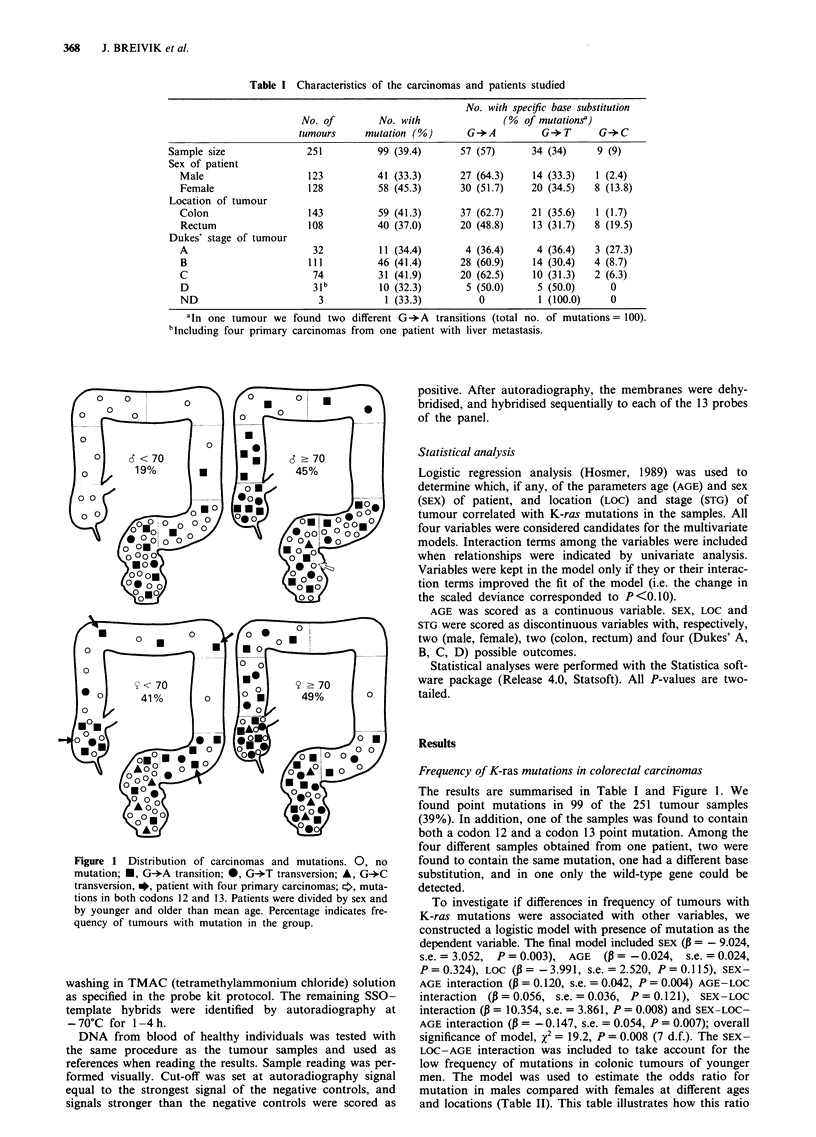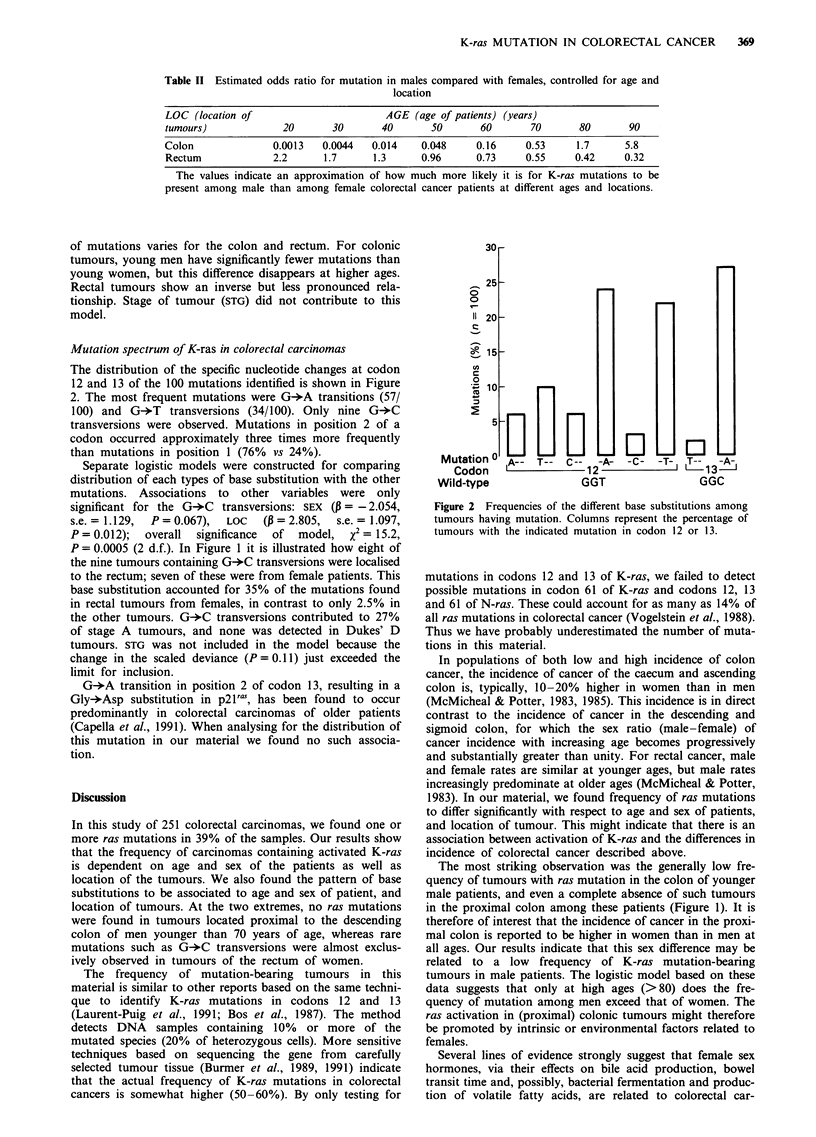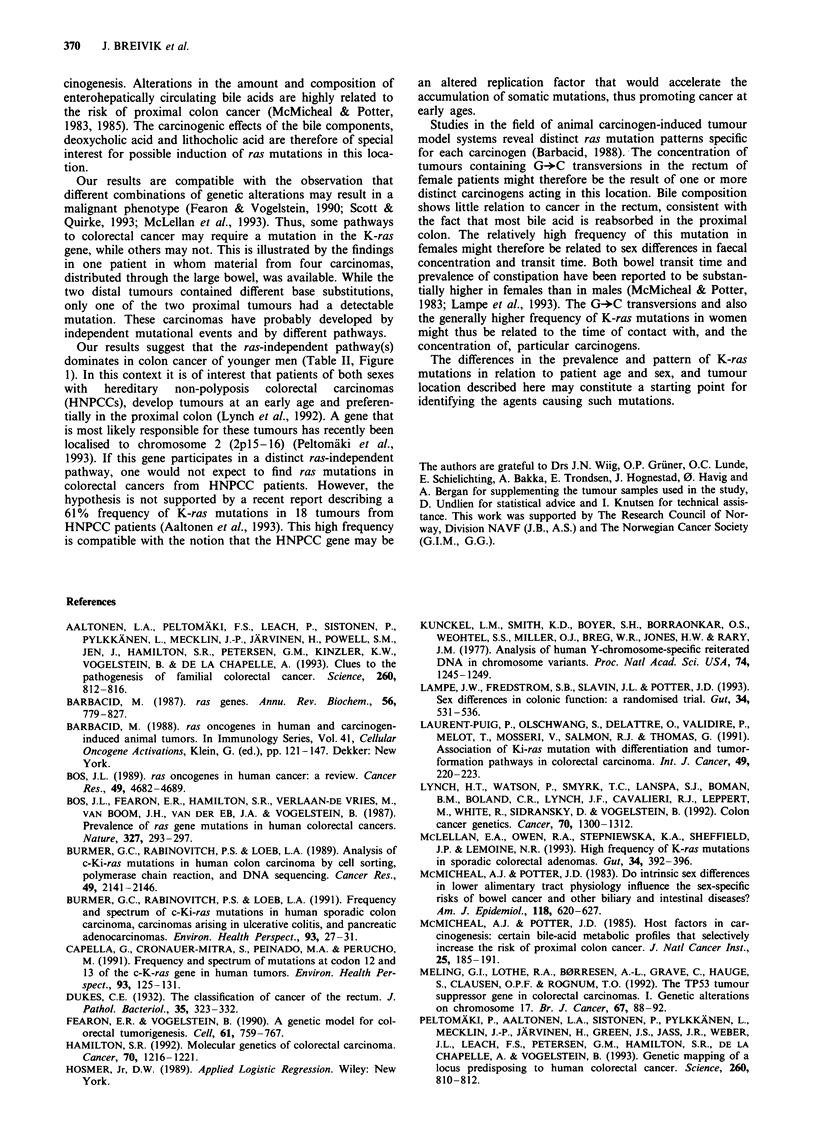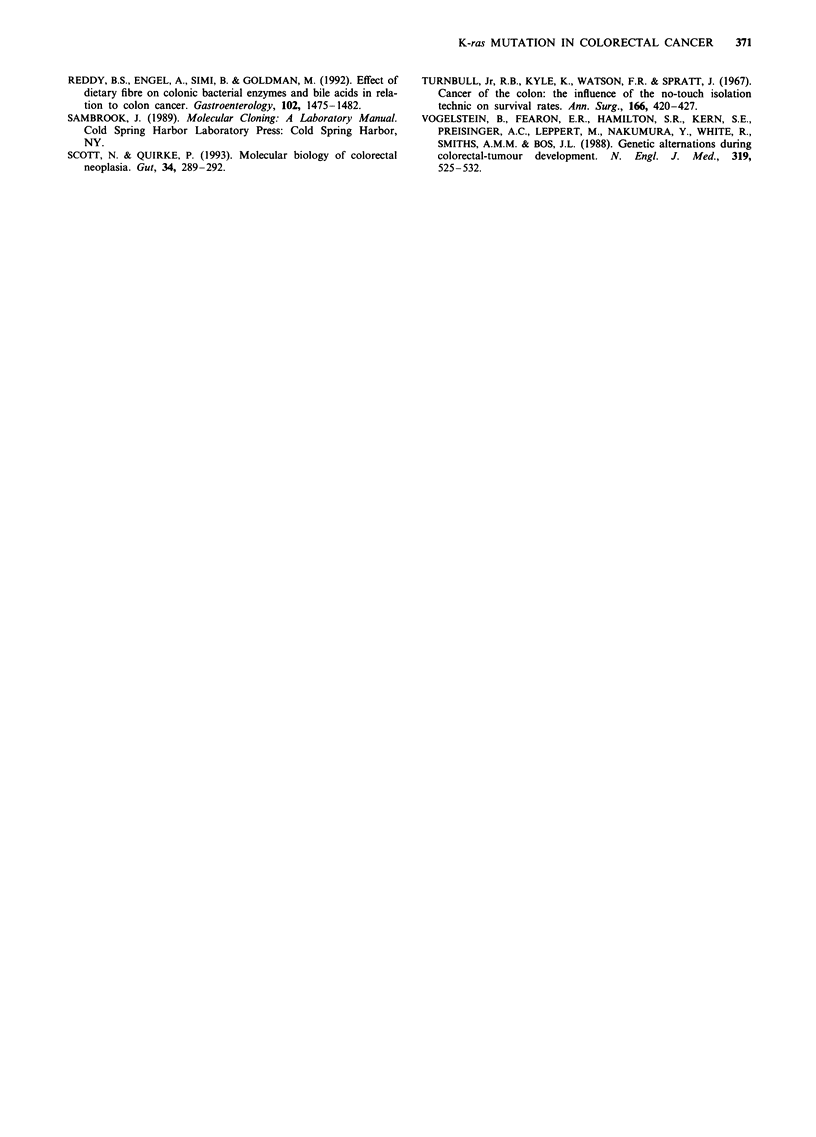# K-ras mutation in colorectal cancer: relations to patient age, sex and tumour location.

**DOI:** 10.1038/bjc.1994.67

**Published:** 1994-02

**Authors:** J. Breivik, G. I. Meling, A. Spurkland, T. O. Rognum, G. Gaudernack

**Affiliations:** Institute of Transplantation Immunology, National Hospital, University of Oslo, Norway.

## Abstract

DNA from 251 primary tumours obtained from 123 male and 125 female Norwegian patients with colorectal carcinoma was analysed for the presence of K-ras point mutations at codons 12 and 13. Mutations were found in 99 (39%) of the samples. The frequency of K-ras mutations was significantly related to age and sex of the patients, and to the location of the tumours (overall: P = 0.008). K-ras mutations were much less frequent in colonic tumours from male than female patients at younger ages (< 40 years, odds ratio < 0.014). The low frequency might indicate that a different, ras-independent, pathway to neoplasia is dominating in the colon of younger males. In contrast, older men had more mutations than older women (e.g. 90 years, odds ratio = 5.8). An inverse but less pronounced relationship was seen for rectal tumours. The type of mutation was found to be associated to sex of patient and location of tumour. G-->C transversions accounted for 35% of the mutations in rectal tumours from females, in contrast to only 2.5% in the rest of the material (P = 0.0005). This may indicate that there are specific carcinogens acting in this location.


Br. J. Cancer (1994), 69, 367-371 © Macmillan Press Ltd., 1994

### K-*ras* mutation in colorectal cancer: relations to patient age, sex and tumour location

J. Breivik[1], G.I. Meling[2], A. Spurkland[1], T.O. Rognum[2] & G. Gaudernack[1]

[1]*Institute of Transplantation Immunology and* [2]*Institute of Forensic Medicine, The National Hospital, University of Oslo, 0027 Oslo, Norway.*

**Summary** DNA from 251 primary tumours obtained from 123 male and 125 female Norwegian patients with colorectal carcinoma was analysed for the presence of K-*ras* point mutations at codons 12 and 13. Mutations were found in 99 (39%) of the samples. The frequency of K-*ras* mutations was significantly related to age and sex of the patients, and to the location of the tumours (overall: $P = 0.008$). K-*ras* mutations were much less frequent in colonic tumours from male than female patients at younger ages (<40 years, odds ratio <0.014). The low frequency might indicate that a different, *ras*-indendent pathway to neoplasia is dominating in the colon of younger males. In contrast, older men had more mutations than older women (e.g. 90 years, odds ratio = 5.8). An inverse but less pronounced relationship was seen for rectal tumours. The type of mutation was found to be associated to sex of patient and location of tumour. G→C transversions accounted for 35% of the mutations in rectal tumours from females, in contrast to only 2.5% in the rest of the material ($P = 0.0005$). This may indicate that there are specific carcinogens acting in this location.

Neoplastic transformation is believed to be the result of accumulation of genetic alterations in a single cell during the life of an individual. Tumour pathogenesis is increasingly understood in terms of damage to critical regulatory genes and the attendant deregulation of biochemical signalling pathways in the cancer cell. The number of genes found to be associated to different malignancies is growing rapidly. Single base substitutions are the genetic change most frequently found (Fearon & Vogelstein, 1990; Capella *et al.*, 1991). The *ras* oncogenes, together with the p53 tumour-suppressor gene, are the genes most consistently found to be mutated in colorectal cancer and a number of other malignancies (Fearon & Vogelstein, 1990; Scott & Quirke, 1993). Single base substitutions in codons 12, 13 or 61 result in an amino acid alteration in the gene product ($p21^{ras}$), activating the oncogenic potential of the *ras* genes. The mutated protein is believed to alter signal transduction pathways in a way that stimulates clonal expansion of the cell (Barbacid, 1987; Bos, 1989).

In sporadic colon carcinomas, K-*ras* mutations have been reported to occur in 40-60% of the cases (Burmer *et al.*, 1989, 1991; Capella *et al.*, 1991), and *ras* mutation appears to be a relatively early event in the adenoma-carcinoma sequence (Fearon & Vogelstein, 1990; Hamilton, 1992). Little is known about the cause and mechanism of activation of *ras* and other oncogenes in the pathogenesis of human cancers. In animal tumour model systems, specific carcinogens have been found to induce distinct mutation patterns in *ras* genes (Barbacid, 1988), but activation of *ras* in human tumours has not been linked to any particular agents. Dietary factors, colonic bacteria and bile composition are, however, related to incidence of colorectal cancer (Reddy *et al.*, 1992), and it is thus believed that mutational events underlying these cancers are caused by stool components.

Stool composition is known to vary with age and sex, and to differ between different segments of the large bowel. We therefore investigated if frequency or type of K-*ras* mutations is related to any of these parameters.

### Materials and methods

### *Patients and tumour samples*

Fresh tissue samples from 251 primary colorectal adenocarcinomas removed during laparotomy of 123 male and 125 female patients were analysed. The patients were between 24 and 94 years at the time of tumour extirpation (mean age 69 years). Four samples were obtained from different primary adenocarcinomas from a female patient with liver metastasis. According to Turnbull's modification of Dukes' classification (Dukes, 1932; Turnbull *et al.*, 1967), the tumours were staged from A to D (Table I). The location of the tumours is illustrated in Figure 1. For comparisons between different anatomical segments, we divided samples obtained from the colon and the rectum into two separate groups. Rectum was defined as the distal 15 cm of the large bowel. Subsegments of the colon are also illustrated.

### *DNA extraction and* in vitro *amplification*

Genomic DNA was extracted from fresh tumour cell suspensions (Meling *et al.*, 1992) using standard methods (Kunckel *et al.*, 1977). A 111 bp fragment of the K-*ras* gene (Genebank accession no. L00045) was amplified using custom synthesised oligonucleotide primers (Genosys Biotechnologies, TX, USA) flanking the codons 12 and 13:

5K0: 5'-ATGACTGAATATAAACTTGT-3'
3K0: 5'-CTCTATTGTTGGATCATATT-3'

Each DNA sample (0.1 μg) was added to 50 μl of PCR buffer (dNTP 0.25 mM each, potassium chloride 50 mM, magnesium chloride 1.5 mM, Tris-HCl 10 mM pH 8.4, gelatin 0.01%). Each primer (20 pmol) and 2.5 units of *Taq* polymerase (AmpliTaq DNA Polymerase, Perkin Elmer, CT, USA) were added. Thirty-five cycles were performed on a DNA thermal cycler (Perkin Elmer). One cycle consisted of 1 min denaturation at 95°C, 1 min annealing at 56°C and 1 min elongation at 72°C. After the last cycle the tubes were kept for 7 min at the elongation temperature.

### *Identification of K-*ras *mutations*

Amplified DNA was slot blotted onto nylon membranes (Bio Trace RP, Gelman Sciences, MI, USA). Samples containing activated *ras* genes were identified using sequence-specific oligonucleotide (SSO) hybridisation with a panel of 13 SSOs. This included SSOs specific for all 12 point mutations in codons 12 and 13 which result in amino acid substitutions, as well as a wild-type probe (Muta-Lyzer oligonucleotide probe panels, Clontech Laboratories, Palo Alto, CA, USA). The probes were radiolabelled ($^{32}P$) according to standard methods (Sambrook, 1989). Hybridisation conditions were as recommended by the manufacturer of the SSOs. Mismatched SSOs were removed from the membrane-bound template by

Correspondence: J. Breivik.
Received 18 May 1993; and in revised form 17 September 1993.

**Table I** Characteristics of the carcinomas and patients studied

| | No. of tumours | No. with mutation (%) | No. with specific base substitution (% of mutations[a]) G→A | G→T | G→C |
|---|---|---|---|---|---|
| Sample size | 251 | 99 (39.4) | 57 (57) | 34 (34) | 9 (9) |
| Sex of patient | | | | | |
| Male | 123 | 41 (33.3) | 27 (64.3) | 14 (33.3) | 1 (2.4) |
| Female | 128 | 58 (45.3) | 30 (51.7) | 20 (34.5) | 8 (13.8) |
| Location of tumour | | | | | |
| Colon | 143 | 59 (41.3) | 37 (62.7) | 21 (35.6) | 1 (1.7) |
| Rectum | 108 | 40 (37.0) | 20 (48.8) | 13 (31.7) | 8 (19.5) |
| Dukes' stage of tumour | | | | | |
| A | 32 | 11 (34.4) | 4 (36.4) | 4 (36.4) | 3 (27.3) |
| B | 111 | 46 (41.4) | 28 (60.9) | 14 (30.4) | 4 (8.7) |
| C | 74 | 31 (41.9) | 20 (62.5) | 10 (31.3) | 2 (6.3) |
| D | 31[b] | 10 (32.3) | 5 (50.0) | 5 (50.0) | 0 |
| ND | 3 | 1 (33.3) | 0 | 1 (100.0) | 0 |

[a]In one tumour we found two different G→A transitions (total no. of mutations = 100). [b]Including four primary carcinomas from one patient with liver metastasis.

**Figure 1** Distribution of carcinomas and mutations. ○, no mutation; ■, G→A transition; ●, G→T transversion; ▲, G→C transversion, ➧, patient with four primary carcinomas; ⇨, mutations in both codons 12 and 13. Patients were divided by sex and by younger and older than mean age. Percentage indicates frequency of tumours with mutation in the group.

washing in TMAC (tetramethylammonium chloride) solution as specified in the probe kit protocol. The remaining SSO–template hybrids were identified by autoradiography at −70°C for 1–4 h.

DNA from blood of healthy individuals was tested with the same procedure as the tumour samples and used as references when reading the results. Sample reading was performed visually. Cut-off was set at autoradiography signal equal to the strongest signal of the negative controls, and signals stronger than the negative controls were scored as positive. After autoradiography, the membranes were dehybridised, and hybridised sequentially to each of the 13 probes of the panel.

*Statistical analysis*

Logistic regression analysis (Hosmer, 1989) was used to determine which, if any, of the parameters age (AGE) and sex (SEX) of patient, and location (LOC) and stage (STG) of tumour correlated with K-*ras* mutations in the samples. All four variables were considered candidates for the multivariate models. Interaction terms among the variables were included when relationships were indicated by univariate analysis. Variables were kept in the model only if they or their interaction terms improved the fit of the model (i.e. the change in the scaled deviance corresponded to $P < 0.10$).

AGE was scored as a continuous variable. SEX, LOC and STG were scored as discontinuous variables with, respectively, two (male, female), two (colon, rectum) and four (Dukes' A, B, C, D) possible outcomes.

Statistical analyses were performed with the Statistica software package (Release 4.0, Statsoft). All *P*-values are two-tailed.

### Results

*Frequency of K-*ras* mutations in colorectal carcinomas*

The results are summarised in Table I and Figure 1. We found point mutations in 99 of the 251 tumour samples (39%). In addition, one of the samples was found to contain both a codon 12 and a codon 13 point mutation. Among the four different samples obtained from one patient, two were found to contain the same mutation, one had a different base substitution, and in one only the wild-type gene could be detected.

To investigate if differences in frequency of tumours with K-*ras* mutations were associated with other variables, we constructed a logistic model with presence of mutation as the dependent variable. The final model included SEX ($\beta = -9.024$, s.e. = 3.052, $P = 0.003$), AGE ($\beta = -0.024$, s.e. = 0.024, $P = 0.324$), LOC ($\beta = -3.991$, s.e. = 2.520, $P = 0.115$), SEX–AGE interaction ($\beta = 0.120$, s.e. = 0.042, $P = 0.004$) AGE–LOC interaction ($\beta = 0.056$, s.e. = 0.036, $P = 0.121$), SEX–LOC interaction ($\beta = 10.354$, s.e. = 3.861, $P = 0.008$) and SEX–LOC–AGE interaction ($\beta = -0.147$, s.e. = 0.054, $P = 0.007$); overall significance of model, $\chi^2 = 19.2$, $P = 0.008$ (7 d.f.). The SEX–LOC–AGE interaction was included to take account for the low frequency of mutations in colonic tumours of younger men. The model was used to estimate the odds ratio for mutation in males compared with females at different ages and locations (Table II). This table illustrates how this ratio

**Table II** Estimated odds ratio for mutation in males compared with females, controlled for age and location

| *LOC (location of tumours)* | *AGE (age of patients) (years)* 20 | 30 | 40 | 50 | 60 | 70 | 80 | 90 |
|---|---|---|---|---|---|---|---|---|
| Colon | 0.0013 | 0.0044 | 0.014 | 0.048 | 0.16 | 0.53 | 1.7 | 5.8 |
| Rectum | 2.2 | 1.7 | 1.3 | 0.96 | 0.73 | 0.55 | 0.42 | 0.32 |

The values indicate an approximation of how much more likely it is for K-*ras* mutations to be present among male than among female colorectal cancer patients at different ages and locations.

of mutations varies for the colon and rectum. For colonic tumours, young men have significantly fewer mutations than young women, but this difference disappears at higher ages. Rectal tumours show an inverse but less pronounced relationship. Stage of tumour (STG) did not contribute to this model.

### *Mutation spectrum of K-*ras *in colorectal carcinomas*

The distribution of the specific nucleotide changes at codon 12 and 13 of the 100 mutations identified is shown in Figure 2. The most frequent mutations were G→A transitions (57/100) and G→T transversions (34/100). Only nine G→C transversions were observed. Mutations in position 2 of a codon occurred approximately three times more frequently than mutations in position 1 (76% *vs* 24%).

Separate logistic models were constructed for comparing distribution of each types of base substitution with the other mutations. Associations to other variables were only significant for the G→C transversions: SEX ($\beta = -2.054$, s.e. = 1.129, $P = 0.067$), LOC ($\beta = 2.805$, s.e. = 1.097, $P = 0.012$); overall significance of model, $\chi^2 = 15.2$, $P = 0.0005$ (2 d.f.). In Figure 1 it is illustrated how eight of the nine tumours containing G→C transversions were localised to the rectum; seven of these were from female patients. This base substitution accounted for 35% of the mutations found in rectal tumours from females, in contrast to only 2.5% in the other tumours. G→C transversions contributed to 27% of stage A tumours, and none was detected in Dukes' D tumours. STG was not included in the model because the change in the scaled deviance ($P = 0.11$) just exceeded the limit for inclusion.

G→A transition in position 2 of codon 13, resulting in a Gly→Asp substitution in p21$^{ras}$, has been found to occur predominantly in colorectal carcinomas of older patients (Capella *et al.*, 1991). When analysing for the distribution of this mutation in our material we found no such association.

### Discussion

In this study of 251 colorectal carcinomas, we found one or more *ras* mutations in 39% of the samples. Our results show that the frequency of carcinomas containing activated K-*ras* is dependent on age and sex of the patients as well as location of the tumours. We also found the pattern of base substitutions to be associated to age and sex of patient, and location of tumours. At the two extremes, no *ras* mutations were found in tumours located proximal to the descending colon of men younger than 70 years of age, whereas rare mutations such as G→C transversions were almost exclusively observed in tumours of the rectum of women.

The frequency of mutation-bearing tumours in this material is similar to other reports based on the same technique to identify K-*ras* mutations in codons 12 and 13 (Laurent-Puig *et al.*, 1991; Bos *et al.*, 1987). The method detects DNA samples containing 10% or more of the mutated species (20% of heterozygous cells). More sensitive techniques based on sequencing the gene from carefully selected tumour tissue (Burmer *et al.*, 1989, 1991) indicate that the actual frequency of K-*ras* mutations in colorectal cancers is somewhat higher (50–60%). By only testing for

**Figure 2** Frequencies of the different base substitutions among tumours having mutation. Columns represent the percentage of tumours with the indicated mutation in codon 12 or 13.

mutations in codons 12 and 13 of K-*ras*, we failed to detect possible mutations in codon 61 of K-*ras* and codons 12, 13 and 61 of N-*ras*. These could account for as many as 14% of all *ras* mutations in colorectal cancer (Vogelstein *et al.*, 1988). Thus we have probably underestimated the number of mutations in this material.

In populations of both low and high incidence of colon cancer, the incidence of cancer of the caecum and ascending colon is, typically, 10–20% higher in women than in men (McMicheal & Potter, 1983, 1985). This incidence is in direct contrast to the incidence of cancer in the descending and sigmoid colon, for which the sex ratio (male–female) of cancer incidence with increasing age becomes progressively and substantially greater than unity. For rectal cancer, male and female rates are similar at younger ages, but male rates increasingly predominate at older ages (McMicheal & Potter, 1983). In our material, we found frequency of *ras* mutations to differ significantly with respect to age and sex of patients, and location of tumour. This might indicate that there is an association between activation of K-*ras* and the differences in incidence of colorectal cancer described above.

The most striking observation was the generally low frequency of tumours with *ras* mutation in the colon of younger male patients, and even a complete absence of such tumours in the proximal colon among these patients (Figure 1). It is therefore of interest that the incidence of cancer in the proximal colon is reported to be higher in women than in men at all ages. Our results indicate that this sex difference may be related to a low frequency of K-*ras* mutation-bearing tumours in male patients. The logistic model based on these data suggests that only at high ages (>80) does the frequency of mutation among men exceed that of women. The *ras* activation in (proximal) colonic tumours might therefore be promoted by intrinsic or environmental factors related to females.

Several lines of evidence strongly suggest that female sex hormones, via their effects on bile acid production, bowel transit time and, possibly, bacterial fermentation and production of volatile fatty acids, are related to colorectal carcinogenesis. Alterations in the amount and composition of enterohepatically circulating bile acids are highly related to the risk of proximal colon cancer (McMicheal & Potter, 1983, 1985). The carcinogenic effects of the bile components, deoxycholic acid and lithocholic acid are therefore of special interest for possible induction of *ras* mutations in this location.

Our results are compatible with the observation that different combinations of genetic alterations may result in a malignant phenotype (Fearon & Vogelstein, 1990; Scott & Quirke, 1993; McLellan *et al.*, 1993). Thus, some pathways to colorectal cancer may require a mutation in the K-*ras* gene, while others may not. This is illustrated by the findings in one patient in whom material from four carcinomas, distributed through the large bowel, was available. While the two distal tumours contained different base substitutions, only one of the two proximal tumours had a detectable mutation. These carcinomas have probably developed by independent mutational events and by different pathways.

Our results suggest that the *ras*-independent pathway(s) dominates in colon cancer of younger men (Table II, Figure 1). In this context it is of interest that patients of both sexes with hereditary non-polyposis colorectal carcinomas (HNPCCs), develop tumours at an early age and preferentially in the proximal colon (Lynch *et al.*, 1992). A gene that is most likely responsible for these tumours has recently been localised to chromosome 2 (2p15-16) (Peltomäki *et al.*, 1993). If this gene participates in a distinct *ras*-independent pathway, one would not expect to find *ras* mutations in colorectal cancers from HNPCC patients. However, the hypothesis is not supported by a recent report describing a 61% frequency of K-*ras* mutations in 18 tumours from HNPCC patients (Aaltonen *et al.*, 1993). This high frequency is compatible with the notion that the HNPCC gene may be an altered replication factor that would accelerate the accumulation of somatic mutations, thus promoting cancer at early ages.

Studies in the field of animal carcinogen-induced tumour model systems reveal distinct *ras* mutation patterns specific for each carcinogen (Barbacid, 1988). The concentration of tumours containing G→C transversions in the rectum of female patients might therefore be the result of one or more distinct carcinogens acting in this location. Bile composition shows little relation to cancer in the rectum, consistent with the fact that most bile acid is reabsorbed in the proximal colon. The relatively high frequency of this mutation in females might therefore be related to sex differences in faecal concentration and transit time. Both bowel transit time and prevalence of constipation have been reported to be substantially higher in females than in males (McMicheal & Potter, 1983; Lampe *et al.*, 1993). The G→C transversions and also the generally higher frequency of K-*ras* mutations in women might thus be related to the time of contact with, and the concentration of, particular carcinogens.

The differences in the prevalence and pattern of K-*ras* mutations in relation to patient age and sex, and tumour location described here may constitute a starting point for identifying the agents causing such mutations.

The authors are grateful to Drs J.N. Wiig, O.P. Grüner, O.C. Lunde, E. Schielichting, A. Bakka, E. Trondsen, J. Hognestad, Ø. Havig and A. Bergan for supplementing the tumour samples used in the study, D. Undlien for statistical advice and I. Knutsen for technical assistance. This work was supported by The Research Council of Norway, Division NAVF (J.B., A.S.) and The Norwegian Cancer Society (G.I.M., G.G.).

### References

AALTONEN, L.A., PELTOMÄKI, F.S., LEACH, P., SISTONEN, P., PYLKKÄNEN, L., MECKLIN, J.-P., JÄRVINEN, H., POWELL, S.M., JEN, J., HAMILTON, S.R., PETERSEN, G.M., KINZLER, K.W., VOGELSTEIN, B. & DE LA CHAPELLE, A. (1993). Clues to the pathogenesis of familial colorectal cancer. *Science*, **260**, 812-816.

BARBACID, M. (1987). *ras* genes. *Annu. Rev. Biochem.*, **56**, 779-827.

BARBACID, M. (1988). *ras* oncogenes in human and carcinogen-induced animal tumors. In Immunology Series, Vol. 41, *Cellular Oncogene Activations*, Klein, G. (ed.), pp. 121-147. Dekker: New York.

BOS, J.L. (1989). *ras* oncogenes in human cancer: a review. *Cancer Res.*, **49**, 4682-4689.

BOS, J.L., FEARON, E.R., HAMILTON, S.R., VERLAAN-DE VRIES, M., VAN BOOM, J.H., VAN DER EB, J.A. & VOGELSTEIN, B. (1987). Prevalence of *ras* gene mutations in human colorectal cancers. *Nature*, **327**, 293-297.

BURMER, G.C., RABINOVITCH, P.S. & LOEB, L.A. (1989). Analysis of c-Ki-*ras* mutations in human colon carcinoma by cell sorting, polymerase chain reaction, and DNA sequencing. *Cancer Res.*, **49**, 2141-2146.

BURMER, G.C., RABINOVITCH, P.S. & LOEB, L.A. (1991). Frequency and spectrum of c-Ki-*ras* mutations in human sporadic colon carcinoma, carcinomas arising in ulcerative colitis, and pancreatic adenocarcinomas. *Environ. Health Perspect.*, **93**, 27-31.

CAPELLA, G., CRONAUER-MITRA, S., PEINADO, M.A. & PERUCHO, M. (1991). Frequency and spectrum of mutations at codon 12 and 13 of the c-K-*ras* gene in human tumors. *Environ. Health Perspect.*, **93**, 125-131.

DUKES, C.E. (1932). The classification of cancer of the rectum. *J. Pathol. Bacteriol.*, **35**, 323-332.

FEARON, E.R. & VOGELSTEIN, B. (1990). A genetic model for colorectal tumorigenesis. *Cell*, **61**, 759-767.

HAMILTON, S.R. (1992). Molecular genetics of colorectal carcinoma. *Cancer*, **70**, 1216-1221.

HOSMER, Jr, D.W. (1989). *Applied Logistic Regression*. Wiley: New York.

KUNCKEL, L.M., SMITH, K.D., BOYER, S.H., BORRAONKAR, O.S., WEOHTEL, S.S., MILLER, O.J., BREG, W.R., JONES, H.W. & RARY, J.M. (1977). Analysis of human Y-chromosome-specific reiterated DNA in chromosome variants. *Proc. Natl Acad. Sci. USA*, **74**, 1245-1249.

LAMPE, J.W., FREDSTROM, S.B., SLAVIN, J.L. & POTTER, J.D. (1993). Sex differences in colonic function: a randomised trial. *Gut*, **34**, 531-536.

LAURENT-PUIG, P., OLSCHWANG, S., DELATTRE, O., VALIDIRE, P., MELOT, T., MOSSERI, V., SALMON, R.J. & THOMAS, G. (1991). Association of Ki-*ras* mutation with differentiation and tumor-formation pathways in colorectal carcinoma. *Int. J. Cancer*, **49**, 220-223.

LYNCH, H.T., WATSON, P., SMYRK, T.C., LANSPA, S.J., BOMAN, B.M., BOLAND, C.R., LYNCH, J.F., CAVALIERI, R.J., LEPPERT, M., WHITE, R., SIDRANSKY, D. & VOGELSTEIN, B. (1992). Colon cancer genetics. *Cancer*, **70**, 1300-1312.

MCLELLAN, E.A., OWEN, R.A., STEPNIEWSKA, K.A., SHEFFIELD, J.P. & LEMOINE, N.R. (1993). High frequency of K-*ras* mutations in sporadic colorectal adenomas. *Gut*, **34**, 392-396.

MCMICHEAL, A.J. & POTTER, J.D. (1983). Do intrinsic sex differences in lower alimentary tract physiology influence the sex-specific risks of bowel cancer and other biliary and intestinal diseases? *Am. J. Epidemiol.*, **118**, 620-627.

MCMICHEAL, A.J. & POTTER, J.D. (1985). Host factors in carcinogenesis: certain bile-acid metabolic profiles that selectively increase the risk of proximal colon cancer. *J. Natl Cancer Inst.*, **25**, 185-191.

MELING, G.I., LOTHE, R.A., BØRRESEN, A.-L., GRAVE, C., HAUGE, S., CLAUSEN, O.P.F. & ROGNUM, T.O. (1992). The TP53 tumour suppressor gene in colorectal carcinomas. I. Genetic alterations on chromosome 17. *Br. J. Cancer*, **67**, 88-92.

PELTOMÄKI, P., AALTONEN, L.A., SISTONEN, P., PYLKKÄNEN, L., MECKLIN, J.-P., JÄRVINEN, H., GREEN, J.S., JASS, J.R., WEBER, J.L., LEACH, F.S., PETERSEN, G.M., HAMILTON, S.R., DE LA CHAPELLE, A. & VOGELSTEIN, B. (1993). Genetic mapping of a locus predisposing to human colorectal cancer. *Science*, **260**, 810-812.

REDDY, B.S., ENGEL, A., SIMI, B. & GOLDMAN, M. (1992). Effect of dietary fibre on colonic bacterial enzymes and bile acids in relation to colon cancer. *Gastroenterology*, **102,** 1475-1482.

SAMBROOK, J. (1989). *Molecular Cloning: A Laboratory Manual.* Cold Spring Harbor Laboratory Press: Cold Spring Harbor, NY.

SCOTT, N. & QUIRKE, P. (1993). Molecular biology of colorectal neoplasia. *Gut*, **34,** 289-292.

TURNBULL, Jr, R.B., KYLE, K., WATSON, F.R. & SPRATT, J. (1967). Cancer of the colon: the influence of the no-touch isolation technic on survival rates. *Ann. Surg.*, **166,** 420-427.

VOGELSTEIN, B., FEARON, E.R., HAMILTON, S.R., KERN, S.E., PREISINGER, A.C., LEPPERT, M., NAKUMURA, Y., WHITE, R., SMITHS, A.M.M. & BOS, J.L. (1988). Genetic alternations during colorectal-tumour development. *N. Engl. J. Med.*, **319,** 525-532.